# Elastohydrodynamics and Kinetics of Protein Patterning in the Immunological Synapse

**DOI:** 10.1371/journal.pcbi.1004481

**Published:** 2015-12-23

**Authors:** Andreas Carlson, L. Mahadevan

**Affiliations:** 1 School of Engineering and Applied Sciences, Kavli Institute for Bionano Science and Technology, and Wyss Institute, Harvard University, Cambridge, United States of America; 2 Departments of Physics, and Organismic and Evolutionary Biology, Harvard University, Cambridge, United States of America; McGill University, CANADA

## Abstract

We propose a minimal mathematical model for the physical basis of membrane protein patterning in the immunological synapse (IS), which encompass membrane mechanics, protein binding kinetics and motion, and fluid flow in the synaptic cleft. Our theory leads to simple predictions for the spatial and temporal scales of protein cluster formation, growth and arrest as a function of membrane stiffness, rigidity and kinetics of the adhesive proteins, and the fluid flow in the synaptic cleft. Numerical simulations complement these scaling laws by quantifying the nucleation, growth and stabilization of proteins domains on the size of the cell. Direct comparison with experiment shows that passive elastohydrodynamics and kinetics of protein binding in the synaptic cleft can describe the short-time formation and organization of protein clusters, without evoking any active processes in the cytoskeleton. Despite the apparent complexity of the process, our analysis shows that just two dimensionless parameters characterize the spatial and temporal evolution of the protein pattern: a ratio of membrane elasticity to protein stiffness, and the ratio of a hydrodynamic time scale for fluid flow relative to the protein binding rate. A simple phase diagram encompasses the variety of patterns that can arise.

## Author Summary

The cellular basis for the adaptive immune response during antigen recognition relies on a specialized protein interface known as the immunological synapse (IS). Understanding the biophysical basis for protein patterning by deciphering the quantitative rules for their formation and motion is an important aspect of characterizing immune cell recognition and thence the rules for immune system activation. We propose a minimal mathematical model for the physical basis of dynamic membrane protein patterning in the IS, which encompass membrane mechanics, protein binding kinetics and motion, and fluid flow in the synaptic cleft. In particular we quantify the nucleation, growth and stabilization of proteins domains. We describe a phase diagram of possible protein patterns by two dimensionless parameters; a ratio of membrane elasticity to protein stiffness, and the ratio of a hydrodynamic time scale for fluid flow relative to the protein binding rate. Direct comparison with experiment suggests that passive processes i.e. viscous fluid flow, elastic membrane bending and protein binding kinetics, can describe the short-time formation and transport of protein clusters, while active cytoskeletal processes enable long-time stabilization of the IS.

## Introduction

Recognition of self or non-self is essential for an effective and functional adaptive immune response. The main players in this process are immune cells (T-lymphocyte cells (T-cells) [1–3], B-cells, natural killer (NK) cells [4] and phagocytes [5, 6] that are constantly on the move scanning surfaces for antigenic peptides on Antigen Presenting Cells (APC). Receptors on the membrane of the immune cells are responsible for sensing and translating information from the extracellular matrix into the cell. Upon antigen recognition the immune cell orchestrates a spatio-temporal organization of its membrane bound proteins into the Immunological Synapse (IS) [7]. Intercellular signaling in a functional IS relates to the formation of large protein domains [2, 3], whereas their formation and the cluster-to-cluster interaction plays an important role in determining the overall cell signaling mechanism. [8].

In the widely studied T-cells, the compartmentalization of membrane-bound protein patterns into different protein domains on the cellular scale leads to the formation of Supra Molecular Activation Clusters (SMACs) [2, 3]. In particular, T-Cell Receptors (TCR) form bonds with the peptide Molecular HistoComplex (pMHC) on the APC, while Leukocyte-Function-Associated antigen-1 (LFA)-integrin on the T-cell bind with Intercellular Adhesion Molecules (ICAM) [3]. Soon (*O*(1 *s*)) [9] after membrane-to-membrane contact sub micron protein clusters are formed that start to translocate (*O*(1 *min*)) [3]. This is followed by long range transport and a concomitant coarsening to form large-scale protein domains at longer times (*O*(40 *min*)) [3, 13]. Observations of the T-cell IS show a central accumulation of TCR-pMHC, surrounded by a donut-shaped preferential protein domain of LFA-ICAM [2, 11] where protein clusters nucleate and act as signaling entities [12–14].

Understanding the biophysical basis for protein patterning by deciphering the quantitative rules for their formation and motion [14] is a first step in characterizing recognition and communication in the immune system. A particularly interesting question in this regard is the role of passive physicochemical processes relative to active motor-driven processes in generating these patterns [15]. Recent experiments suggest that early on during the process, active processes may not be important, and it is only later that the protein pattern in the T-cell membrane is subject to cytoskeletally generated centripetal transport [16–23]. The question of characterizing the mechanics of the IS patterns has led to a range of mathematical models that take one of two forms: those that treat the system as a collection of discrete units [25–29] or as a continuum [30, 32, 33]. While these models are capable of explaining the spatial patterning seen in the IS, they all neglect the fluid flow in the synaptic cleft and thus rely on ad-hoc assumptions for the characteristic time scales over which the patterns form, and use approaches based on gradient descent [30, 32–34] or stochastic variations of energy minimization of the membrane coupled to protein kinetics [25–28].

Here, we provide a description of the passive responses in the IS which includes the mechanical forces due to stretching and bending of the cell membrane which are driven by protein attachment and fluid flow, which itself causes flow of the trans-membrane proteins. This requires that we integrate cell membrane bending and tension, viscous flow in the synaptic cleft and protein attachment-detachment kinetics, and allows us to capture the essential spatiotemporal protein dynamics (nucleation, translation and coalescence of protein clusters) during the formation of SMACs. Furthermore, we show that our description of the passive dynamics in the IS implies that the slow dynamics of fluid flow can limit the rate of protein patterning, without evoking any active cytoskeletal processes.

## Methods

### Membrane mechanics

**Fig 1:**
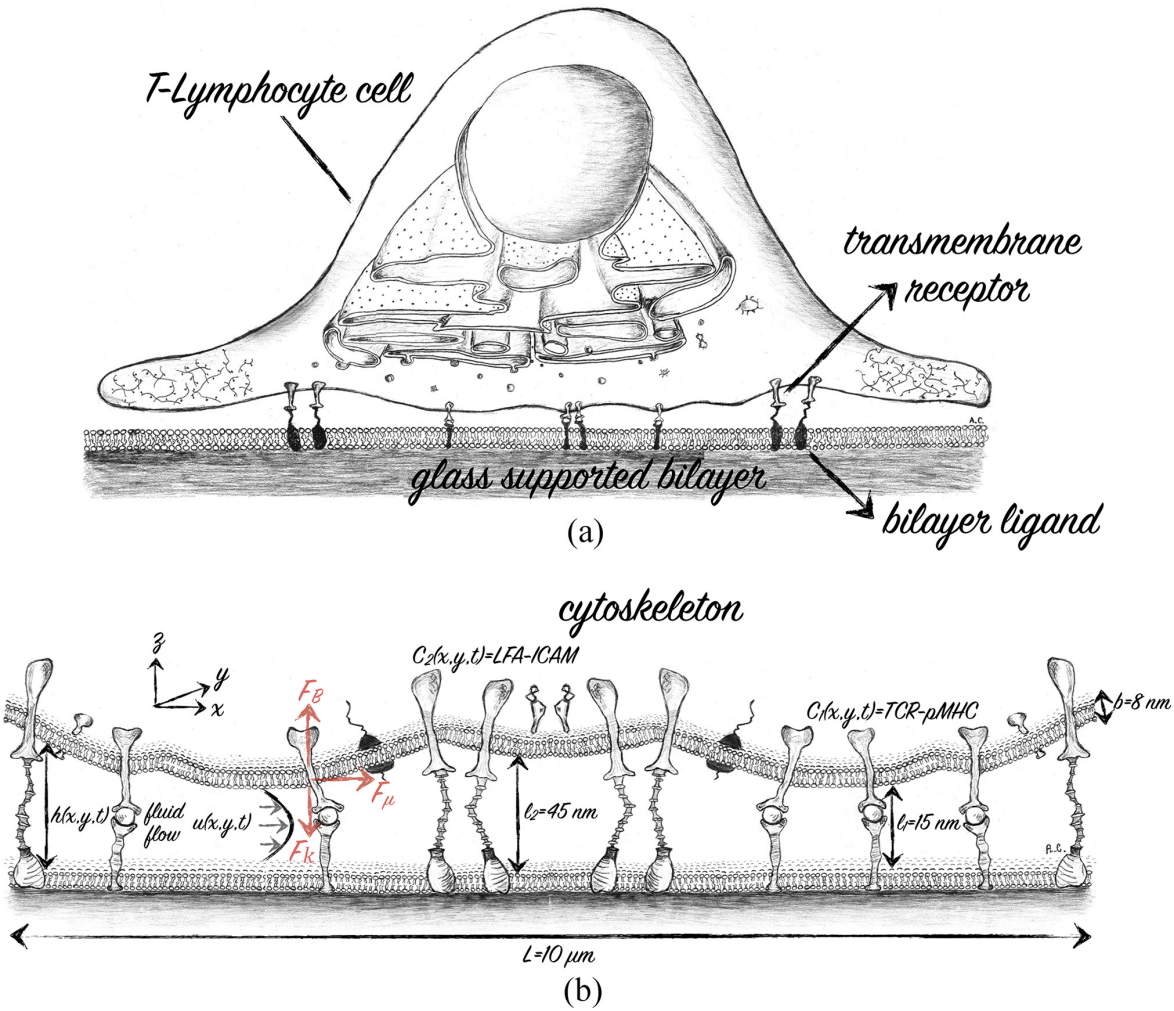
(a) Sketch of the interaction between a T-lymphocyte cell (T-cell) and a supported antigen-seeded bilayer. The two membranes are separated by transmembrane receptors bound to ligands in the bilayer. (b) Close-up schematic view of the synaptic cleft formed between the T-cell membrane and the glass supported bilayer. The cell membrane has a thickness *b* ≈ 8 *nm* and the membrane gap height is given by *h* = *h*(*x*, *y*, *t*). The trans-membrane receptors form bonds with the ligands in the bilayer with lengths and concentrations, *TCR* − *pMHC* ≈ 15 *nm*, *C*_1_(*x*, *y*, *t*), and *LFA* − *ICAM* ≈ 45 *nm*, *C*_2_(*x*, *y*, *t*). During protein bond formation and depletion, the cell membrane deforms generating a viscous flow **u**(*x*, *y*, *z*) in the membrane gap. The flow generates a viscous frictional force *F*_*μ*_ parallel with the glass supported bilayer that acts on the cell membrane and the transmembrane proteins and thus affects their motion. Any deformation of the membrane generates a restoring elastic bending force *F*_*B*_, while the deformation of the TCR-pMHC and LFA-ICAM bonds generates a spring force *F*_*κ*_.

In Fig 1 we illustrate the interaction between a T-cell and an antigen seeded bilayer, which mimics the most commonly used experimental setup [3, 20, 21, 23], and describes the components in the mathematical model described below. Once the T-cell is close to the bilayer (Fig 1) the membrane-bound receptors form adhesive bonds with their ligand counterparts in the bilayer, which pull the membranes together and squeezing the fluid out of the cleft.

When these two types of receptors form bonds with ligands, they get compressed or stretched. We assume that their spring stiffnesses $\kappa_{i}=\frac{l_{1}}{l_{i}}\kappa$ are inversely proportional to the protein length *l*_*i*_ that may vary among different protein types [31]. The subscript *i* = 1 corresponds to the TCR-pMHC complex and *i* = 2 corresponds to the LFA-ICAM complex. *C*_*i*_ = *C*_*i*_(*x*, *y*, *t*) is the number of attached proteins per surface area (associated with at the total equilibrium receptor density *C*_0_), their deformation creates a local pressure ∼ *κ*_*i*_ *C*_*i*_(*x*, *y*, *t*)(*l*_*i*_ − *h*). This pressure deforms the cell membrane, approximated here as a bilayer with a bending stiffness $B_{m}=\frac{Eb^{3}}{12(1-\nu^{2})}$, with *E* the Young’s modulus, *b* the membrane thickness and *ν* the Poisson ratio (see S1 Text), and a mechanical response quantified by

$$p\left(x,y,t\right)=B_{m}\nabla^{4}h+\kappa C_{1}\left(h-l_{1}\right)+\kappa \frac{2l_{1}}{l_{2}}C_{2}\left(h-l_{2}\right)$$ (1)

where *p* = *p*(*x*, *y*, *t*) is the pressure along the membrane, and *h* = *h*(*x*, *y*, *t*) is the height of the fluid-filled synaptic cleft. We focus here on the limit when membrane bending dominates, but we show in the S3 Fig that the influence of membrane tension smooths some of the small scale pattern features. Active cytoskeletal forces would appear as additional source terms in *p*, but has been neglected below as we focus on the passive dynamics.

### Hydrodynamics

Any membrane deformation initiates fluid motion and give rise to hydrodynamic forces in the synaptic cleft, which consequently affects the membrane dynamics. In typical experiments, the synaptic pattern has a lateral size *L* comparable to the cell size (≈ 10 *μm*), while the cleft has a height comparable to size of the longest protein bond (*l*_2_ = 45 *nm*). Thus the aspect ratio of the IS is small *l*_2_/*L* ≪ 1. When combined with the fact that at these small length scales, the flow in the synaptic cleft is viscously dominated, we may use lubrication theory [36] to simplify the equations governing fluid flow. Under the assumption of local Poiseuille flow [36] we can derive a single non-linear scalar partial differential equation for the thin film height *h*(*x*, *y*, *t*) [37] similar to that seen in other elastohydrodynamic phenomena [38–40]

$$\begin{array}{cc} & \frac{\partial h}{\partial t}=\nabla \cdot (\frac{h^{3}}{12\mu }\nabla p),i.e.\\ & \frac{\partial h}{\partial t}=\nabla \cdot (\frac{h^{3}}{12\mu }\nabla \;(B_{m}\nabla^{4}h+\kappa C_{1}\left(h-l_{1}\right)+\kappa \frac{l_{1}}{l_{2}}C_{2}\left(h-l_{2}\right))), \end{array}$$ (2)

where Eq 2 follows by using Eq 1 for pressure (*p*), where *μ* is the fluid viscosity. We note that the presence of proteins and other polymers in the membrane gap can lead to the formation of a porous structure that impedes flow and leads to a Darcy-like regime rather than a Poiseuille-like regime. In this situation, we expect the flow to be determined by the relation $u\approx \frac{-K_{p}}{\mu }\nabla p$, where *K*_*p*_ is the effective hydraulic permeability in the gap; when *K*_*p*_ = *h* ^2^ we recover the Poiseuille relationship used in Eq 2. Here, we limit ourselves to the Poiseuille-form, noting that many qualitative features of our results will carry over to the Darcy regime as well. Here, we have also neglected the effects of fluid permeation across the membrane in the absence of experimental evidence for this. Finally we have neglected thermal fluctuations of the membrane since these will be strongly damped by enthalpic protein binding.

### Protein kinetics

We only follow the dynamics of the membrane-bound proteins that can bind and unbind from their complementary ligands, which is equivalent to stating that the number of these proteins involved in the binding kinetics is large compared to the free proteins in the cytoplasm. In the membrane we assume that the total number of membrane-bound proteins per unit area is constant and given by *C*_*i*,0_, where *i* = 1 corresponds to TCR and *i* = 2 corresponds to LFA. Of these, the number density of bound receptors is denoted by *C*_*i*_(*x*, *y*, *t*). Their dynamics can be described mathematically by a reaction-convection-diffusion equation, which accounts for their diffusion and transport in addition to the binding and detachment, and in dimensional form reads

$$\frac{\partial C_{i}}{\partial t}=\frac{hl_{i}}{\mu }\nabla P\cdot \nabla C_{i}+\nabla \cdot (D_{i}\nabla C_{i}+\frac{k_{b}TD_{i}}{\mu }\left(C_{i}\nabla h\left(h-l_{i}\right)\right))+\left(C_{i,0}-C_{i}\right)K^{on}\left(l_{i}\right)-C_{i}K^{off}\left(l_{i}\right).$$ (3)

The first term on the right side is an advective term due to the fluid flow in the synaptic cleft driven by local pressure gradients associated with membrane deformation. The second term is a membrane protein flux due to molecular diffusion *D*_*i*_∇*C*_*i*_, where the diffusion coefficient *D*_*i*_ = (*l*_1_/*l*_*i*_)*D* is assumed to be inversely proportional to the protein length following the Stokes-Einstein equation. Alternatively, the membrane diffusivity can be influenced by the membrane anchors, but our results are fairly insensitive to the molecular diffusion term (see SI) and we ignore them here. The third term on the right side is a drift in response to membrane deformation at a rate $\frac{k_{B}TD_{i}}{\mu }\nabla (C_{i}\nabla h(h-l_{i})$ [30, 34], where *K*_*B*_ *T* is the thermal energy. The last two terms correspond to receptor binding at a rate (*C*_*i*,0_ − *C*_*i*_)*K* ^*on*^(*l*_*i*_) and unbinding at a rate *C*_*i*_ *K* ^*off*^(*l*_*i*_). The kinetic rates *K* ^*on*^(*l*_*i*_) and *K* ^*off*^(*l*_*i*_) are described in terms of the mean first passage time over an energy barrier [41, 42], with a distribution centered around the natural protein length (*l*_*i*_) and being a function of *l*_*i*_/*l*_2_ − *h*, given by

$$\begin{array}{cc} \text{Bond}\;\text{formation}:\;\;\; & K^{on}\left(l_{i}\right)=\frac{1}{\tau_{k}}\exp\left(-{\left(\frac{\frac{l_{i}}{l_{2}}-\frac{h}{l_{2}}}{\frac{\sigma_{on}l_{i}}{l_{2}}}\right)}^{2}\right)\\ \text{Bond}\;\text{depletion}:\;\;\; & K^{off}\left(l_{i}\right)=\frac{1}{3\tau_{k}}\exp\left(-{\left(\frac{\frac{l_{i}}{l_{2}}-\frac{h}{l_{2}}}{\frac{\sigma_{off}l_{i}}{l_{2}}}\right)}^{2}\right), \end{array}$$ (4)

where *τ*_*k*_ is the kinetic time. To favor protein binding for *h* ∼ *l*_*i*_, we assume that proteins lose their bonds three times slower (3*τ*_*k*_) [27] than the rate at which they form. Alternatively, if we assume that the off-rate increases with spring tension, so that proteins would unbind as *h* ≪ *l*_*i*_ and *h* ≫ *l*_*i*_ and in its simplest form given by a constant off-rate (*σ*_*off*_ = ∞) in Eq 4 that produce similar results (see SI). Although the exact form of these rates are not known, experiments show that the the different protein pairs form non-overlapping patterns [2, 3, 20], which we mimic via the choice of the width of the kinetic distributions *σ*_*on*_ = 0.2 and *σ*_*off*_ = 0.6 [35]. Narrowing the distributions generates wider protein free areas that separate TCR-pMHC and LFA-ICAM rich regions. In contrast, increasing the distribution widths make the different protein species overlap, which is unrealistic. All together, our model focuses on protein transport due to physicochemical processes driven by protein binding, fluid flow and membrane deformation and neglects the role of active cytoskeleton dynamics in the cell e.g. polarized release of T-cell-receptor-enriched microvesicles [24], endocytosis and exocytosis of proteins [5].

### Dimensional parameters

The material properties of the cell, the fluid and the proteins that are relevant to the IS and needed as input into Eqs 1–4 are summarized in Table 1 as reported in previous work in the literature.

**Table 1:** Description of the material parameters that appear in Eqs 1–4.

**Description**	**Notation**	**Reference**
Fluid viscosity	*μ* = 4 × 10^−2^ *Pa* ⋅ *s*	
Cell membrane Young’s modulus	*E* = (0.08 − 80) × 10^6^ *Pa*	
Membrane thickness	*b* = 8 × 10^9^ *m*	
Poisson ratio	*ν* = 0.5	[52]
Bending modulus	$B_{m}=\frac{Eb^{3}}{12(1-\nu^{2})}=4.5\times (10^{-21}-10^{-19})$ J	[30, 32]
		[52]
Protein stiffness (Hookean spring)	*κ* = 1.2 × 10^−6^ *N*/*m*	[29, 30]
		[34]
Equilibrium number density TCR	*C*_1,0_ = *C*_0_ = 2 × 10^14^ *m* ^−2^	[3]
Equilibrium number density LFA	*C*_2,0_ = 2 × *C*_0_ = 4 × 10^14^ *m* ^−2^	[3]
Natural TCR-pMHC length	*l*_1_ = 15 *nm*	[21]
Natural LFA-ICAM length	*l*_2_ = 45 *nm*	[21]
Membrane protein diffusion coefficient	*D* = 5 × 10^−13^ *m*/*s* ^2^	[50, 51]
Kinetic on-rate	*τ*_*k*_ = *τ*_1_ = *τ*_2_ = 1.1 × (10^−5^ − 10^−1^)*s*	
Kinetic off-rate	$\tau_{off}^{c}=\tau_{off}^{g}=\tau_{k}/3$ s	[27]
Cell diameter	*L* = 10 *μm*	[3]
Hydrodynamic time scale	$\tau_{\mu }=\frac{\mu }{C_{0}\kappa l_{2}}=3.7\times 10^{-3}$ s	
Thermal energy	*k*_*B*_ *T* = 4.34 × 10^−21^ *J*	
Distribution width on-rate	*σ*_*on*_ = 0.2	
Distribution width off-rate	*σ*_*off*_ = 0.6	
Pressure scaling	*p*_0_ = *C*_0_ *κl*_2_ = 10.8 *Pa*	

### Dimensionless numbers

It is natural to scale the horizontal length scales using the cell size, i.e. [*x*, *y*] ∼ *L*, the height of the synaptic cleft using the typical protein length i.e. *h* ∼ *l*_2_, the pressure by the local receptor force/area, i.e. *p* ∼ *C*_0_ *κl*_2_ ≡ *p*_0_, and time by a viscous time, i.e. $\tau_{\mu }=\frac{\mu }{C_{0}\kappa l_{2}}$.

In Eqs 1–4, the use of the scaled variables *p*(*x*, *y*, *t*) = p*(*x*, *y*, *t*)*p*_0_ = p*(*x*, *y*, *t*)*C*_0_ *κl*_2_, *h*(*x*, *y*, *t*) = *h*(*x*, *y*, *t*)*l_2_, *x* = x*L, *y* = y*L, *t* = t*τ_*μ*_, *C*_*i*_(*x*, *y*, *t*) = *C*_*i*_(*x*, *y*, *t*)*C_0_ yields six non-dimensional numbers that govern the dynamics of protein patterning, as shown in Table 2: $B=\frac{B_{m}}{\kappa C_{0}L^{4}}$ is the ratio of pressure generated by membrane bending and the protein spring pressure, *l*_1_/*l*_2_ is the ratio between the natural length of the proteins which is approximately 1/3, *l*_2_/*L* is the aspect ratio of the membrane gap, $Pe=\frac{L^{2}C_{0}\kappa l_{2}}{D\mu }$ is the ratio between advection and diffusion, $M=\frac{D\mu }{k_{b}TC_{0}l_{2}}$ is the ratio between protein diffusion and protein sliding mobility, $\tau =\frac{\tau_{\mu }}{\tau_{k}}=\frac{\mu }{\tau_{k}C_{0}\kappa l_{2}}$ is the ratio between the local hydrodynamic time *τ*_*μ*_ and the kinetic time *τ*_*k*_ (Table 1). As we will show, our results are insensitive to variations in *Pe*, *M* and initial conditions (see SI), and only the dimensionless numbers *B* and *τ* control the qualitative aspects of our phase space of patterns.

**Table 2:** By substituting the scaled variables in Eqs 1–4; *p*(*x*, *y*, *t*) = p*(*x*, *y*, *t*)*p*_0_ = p*(*x*, *y*, *t*)*C*_0_ *κl*_2_, *h*(*x*, *y*, *t*) = *h*(*x*, *y*, *t*)*l_2_, *x* = x*L, *y* = y*L, *t* = t*τ_*μ*_, *C*_*i*_(*x*, *y*, *t*) = *C*_*i*_(*x*, *y*, *t*)*C_0_ gives the non-dimensional numbers above. These non-dimensional numbers characterize the relative influence of membrane mechanics, protein kinetics, geometry and hydrodynamics.

**Description**	**Non-dimensional number**
Membrane bending/protein stretching	$B=\frac{Eb^{3}}{12(1-\nu^{2})\kappa C_{0}L^{4}}=\frac{B_{m}}{\kappa C_{0}L^{4}}=2\times (10^{-7}-10^{-9})$
Aspect ratio membrane height/length	$\frac{l_{2}}{L}=4.5\times 10^{-3}$
Protein aspect ratio TCR-pMHC/LFA-ICAM	$\frac{l_{1}}{l_{2}}=\frac{l_{1}}{l_{2}}=\frac{1}{3}$
Diffusive/advective time scale	$Pe=\frac{L^{2}C_{0}\kappa l_{2}}{D\mu }=5\times 10^{4}$
Protein sliding mobility/protein diffusion	$M=\frac{k_{b}TC_{0}l_{2}}{D\mu }=2.0$
Hydrodynamic/kinetic time scale	$\tau =\frac{\tau_{\mu }}{\tau_{k}}=\frac{\mu }{\tau_{k}C_{0}\kappa l_{2}}=3\times (10^{-3}-10)$

The variations in two important dimensionless numbers *B* and *τ* can be used to capture the potential variations in the membrane properties and/or the protein biochemistry across different experiments. In particular, the membrane properties depends on its composition, where the presence of inclusions e.g. cholesterol, peptides, proteins, can alter its stiffness. *τ* depends on the fluid in the synaptic cleft and the biochemistry of protein binding. In particular, if *τ* > 1 bonds form rapidly relative to the time for fluid flow in the cleft which is then rate limiting, and conversely when *τ* < 1, fluid flow is fast relative to bond formation which is then rate limiting.

### Scaling laws—length

Two characteristic lengths are observed in the IS, the micro-cluster scale *l*_*c*_ and the large domain scale *L*. From Eq 1 we derive a scaling law for the cluster size, by balancing the spring pressure and bending pressure $B_{m}l_{2}/{l_{c}}^{4}\approx C_{0}l_{2}$ that leads to

$$l_{c}\approx {\left(\frac{B_{m}}{C_{0}\kappa }\right)}^{\frac{1}{4}}.$$ (5)

For the simulated *B*_*m*_ (SI) $l_{c}\approx {\left(\frac{B_{m}}{C_{0}\kappa_{1}}\right)}^{\frac{1}{4}}=70-200\;nm$ i.e. in dimensionless units $l_{c}^{*}=B^{\frac{1}{4}}\approx 0.02-0.06$ for *B* ∈ [10^−9^, 10^−7^], qualitatively consistent with experimental observations [20, 21].

### Scaling laws—time

Protein patterning at the micro-cluster (*l*_*c*_) size occurs on short time scales (*τ*_*c*_), while patterning at the cell scale (*L*) occurs on long time scales (*τ*_*L*_). Fluid continuity and force balance embodied in Eq 2 yields a short time scale *τ*_*c*_ corresponding to drainage on the micro-cluster scale *l*_*c*_, given by

$$\tau_{c}=12{\left(\frac{l_{c}}{l_{2}}\right)}^{2}\tau_{\mu }=12{\left(\frac{B_{m}}{C_{0}\kappa l_{2}^{4}}\right)}^{\frac{1}{2}}\frac{\mu }{C_{0}\kappa l_{2}}.$$ (6)

Substituting in parameter values yields *τ*_*c*_ ≈ 0.1–1 *s* i.e. in dimensionless time units $\tau_{c}^{*}=12{\left(\frac{l_{c}}{l_{2}}\right)}^{2}\approx 24-240$ (see SI). Fluid drainage on the cellular scale *L* yields a long time scale given by

$$\tau_{L}=12{\left(\frac{L}{l_{2}}\right)}^{2}\tau_{\mu }=12{\left(\frac{L}{l_{2}}\right)}^{2}\frac{\mu }{C_{0}\kappa l_{2}}.$$ (7)

Substituting parameter values yields *τ*_*L*_ ≈ 40 *min* i.e. in dimensionless units $\tau_{L}^{*}\approx 5\times 10^{4}$.

### Computational methodology and boundary conditions

To solve the nonlinear system of Eqs 1–4, we numerically discretize these with a finite element method (see S1 Text) in two-dimensions, which gives the membrane topography in three-dimensions. For consistency with experimental observations, the simulations are performed in a circular domain that capture the central region of the cell-to-cell contact, which is assumed not to be influenced by the motion of the cell leading edge. At the edge of the IS the membrane is assumed to be torque free with no bending moment (∇^2^ *h* = 0) and at a constant pressure (*p* = 0), which allows fluid flux through the boundary. The membrane is pinned at the edge (*h* = 0.5*l*_2_) and the equilibrium number of proteins per membrane area at that given height (*C*_1_ = *C*_2_ = 0.01*C*_0_) see [35] and S1 Text and S1 Fig for details. The membrane is initialized with six small Gaussian shaped bumps of different widths (≈ 0.1*L*) and amplitude ((0.075–0.1)*l*_2_). Additional information about the numerical method [48], [49], parameter sensitivity and alternative boundary conditions are in the SI.

## Results

Within the phase space described by *B* and *τ*, we start by considering a cell that has a stiffness that scales with the thermal energy *B*_*m*_ ≈ *k*_*B*_ *T* and binding rates that are similar to those reported in experiments [3] ≈ 10^−4^ *Ms*, with an association constant ≈ 0.1*M* ^−1^ giving *τ*_*k*_ ≈ 10^−5^ s. We note that the hydrodynamic time scale is larger *τ*_*μ*_ ≈ 3 × 10^−3^ *s* than *τ*_*k*_ suggesting that the IS dynamics is rate limited by the fluid flow i.e. *τ* ≫ 1, which we verify below.

**Fig 2:**
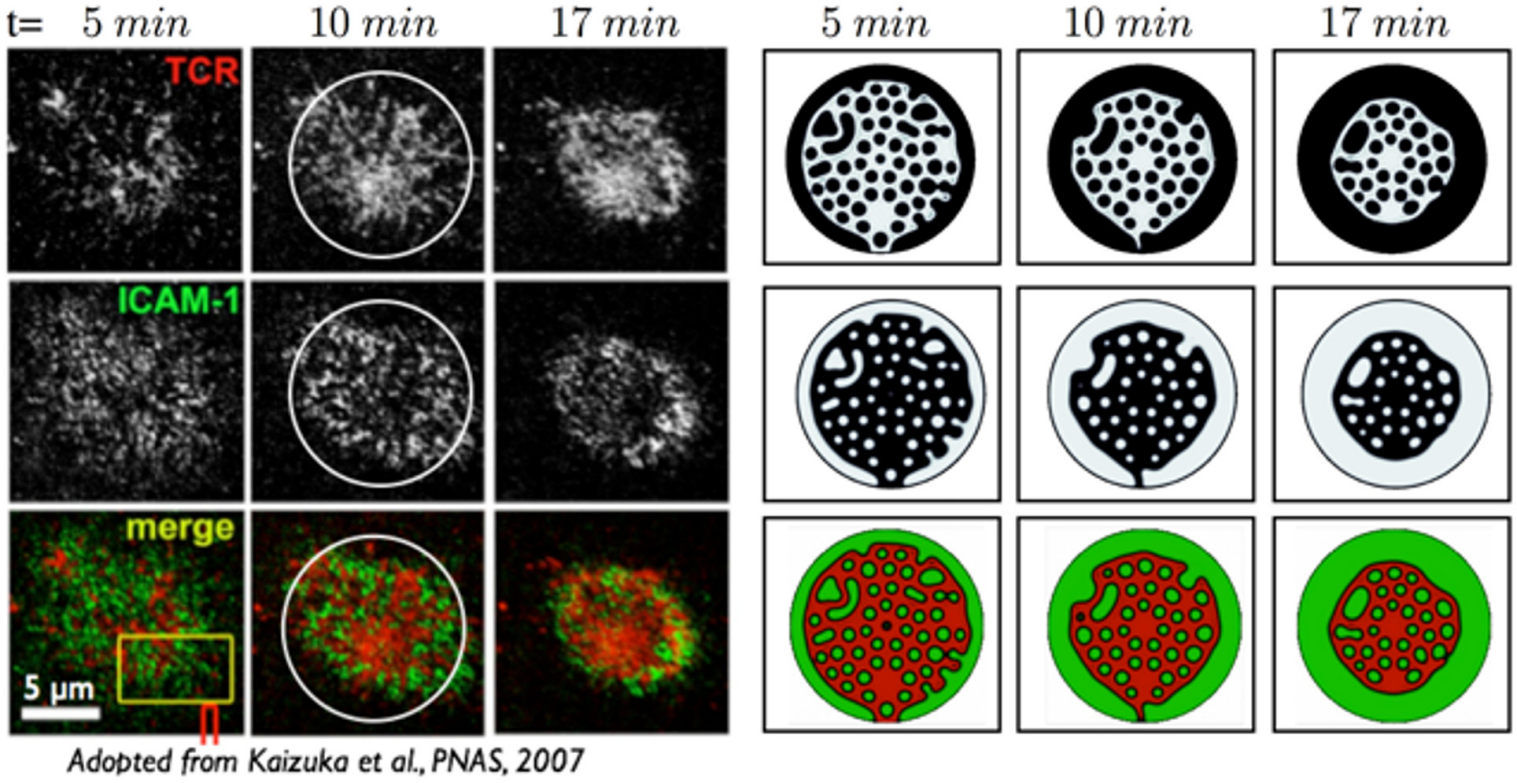
Comparison between an experimental (left) and numerical (right) realization of the TCR-pMHC and LFA-ICAM protein patterning dynamics in the IS. The simulations are based on Eqs 1–4 allowing fluid flux at the edge of the IS, where the height and number of proteins per membrane area is fixed. In the experiment a T-cell interacts with an antigen seeded lipid bilayer [20]. The upper row shows the density of bonded TCR-pMHC, the middle row the bonded LFA-ICAM proteins and the last row their union. The right panel shows the numerical simulation with *B* = 2 × 10^−9^ and *τ* = 15 with non-dimensional times ${\left(\frac{h_{0}}{L}\right)}^{2}\times t=[1.7,\;3.3,\;5.7]$. All other non-dimensional numbers are reported in Table 2. At short-times, protein clusters nucleate on the membrane, with a dynamics given by the interplay between membrane mechanics, protein kinetics and fluid flow. At late times protein clusters interact and coalesce into large spatial patterns that mimic pSMAC and cSMAC structures. A “donut shaped” LFA ring surrounds a dense TCR region at the center of the synapse at late times.

In Fig 2 we show the time evolution of the IS for these parameters (*B* = 2 × 10^−9^, *τ* = 15) and note that the qualitative behavior of our model is consistent with the observed asymmetric IS dynamics [3, 11, 14, 20, 43] (see S1 Movie), and recapitulates the protein aggregation of dense non-overlapping regions of TCR-pMHC and LFA-ICAM, which vary with time. At short times dispersed micron-sized protein clusters nucleate on the membrane, with a characteristic cluster size ≈ 1*μm* (containing ≈ 160 proteins). These protein clusters are transported by the centripetal fluid flow generated by membrane deformation. At long times, we see the appearance of larger spatial protein domains, with a “donut-shaped” LFA-ICAM structure (peripheral SMAC) surrounding a dense central domain of TCR-pMHC (central SMAC) (Fig 2). This similarity is particularly striking since we did not evoke any active processes. We note that our results are also in concordance with recent experiments on latrunculin treated cells [13], wherein disrupting the actin cytoskeleton does not change the early-stage patterns.

**Fig 3:**
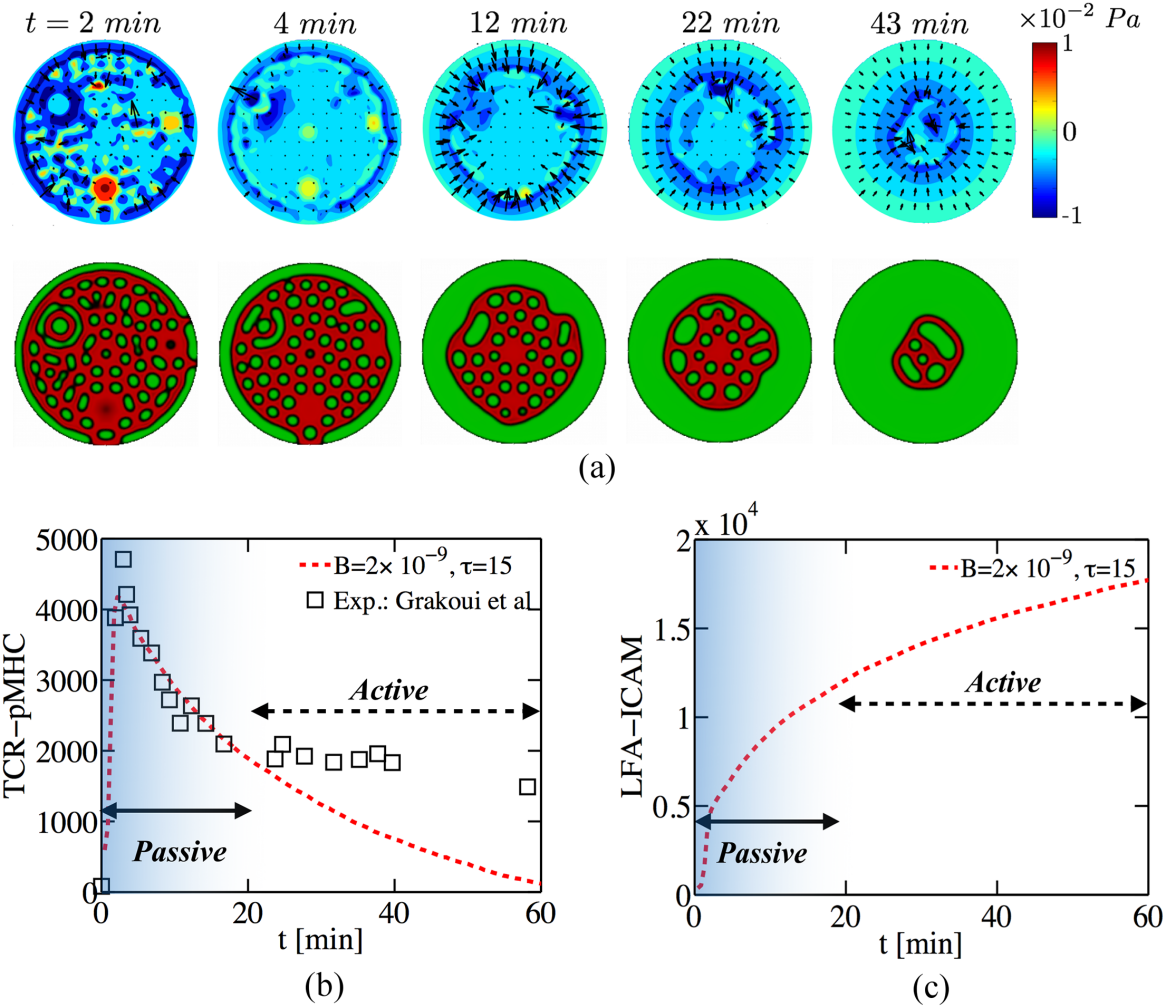
(a)-(c) Simulation of Eqs 1–4 with *B* = 2 × 10^−9^ and *τ* = 15 and the other dimensionless numbers are reported in Table 2. (a) Contour plots of the time history of the pressure (*p*), along with the velocity (−*h* ^2^∇*p*). The second row shows the corresponding protein pattern of TCR-pMHC and LFA-ICAM, see Fig 3 for color scale. At short times (*t* < 4 *min*) the nucleation and coalescence of protein domains generates a local flow field. At late times (*t* ≥ 12*min*) a global centripetal flow is generated that “compress” the TCR cluster radially generating a “bulls-eye”-like protein pattern, which becomes unstable at *t* ≈ 60*min*. (b-c)The total number of attached receptors of (b) TCR-pMHC and (c) LFA-ICAM. (b-c) Direct comparison between the total number of attached TCR in the IS in simulation and in experiment [3] shows that the results are in good agreement for *t* < 20 *min*. This suggests that passive dynamics suffices to describe the short-time formation and organization of protein domains while the long-time IS dynamics and its stability is likely controlled by active processes.

To illustrate how these transport processes are correlated with domain coarsening, we show the pressure and velocity fields in Fig 3a. At short times (*t* < 4 *min*) the nucleation and coalescence of protein domains at a length scale ≈ *l*_*c*_ generates a local flow field, while at long times (*t* > 12 *min*) the flow occurs over a global length scale ≈ *L* wherein the centripetal flow moves the clusters to the center of the domain and coarsens the protein pattern. In Fig 3b we directly compare the dynamics of the TCR clusters in the simulation with experiments [3]. With increasing time, the number of attached TCR rapidly increases upon first contact as micro clusters nucleate. A distinct peak in the number of attached TCR is observed around *t* ≈ 5 *min* in Fig 3b, followed by a decay in the number of attached receptors over longer times. The agreement with experiments for *t* < 20 *min* is striking since no active processes are evoked and suggests that the slow dynamics of fluid drainage in the synaptic cleft limits the rate of protein patterning during the early stages of IS dynamics.

At longer times (*t* > 20 *min*) the results of the simulation and experiments deviate from each other, indicating an important role for active processes to stabilize the dynamical synapse. Over this period (≈ 60 *min*), a distinctive feature in experiment [3] is the appearance of a stable dense circular region of TCR-pMHC surrounded by a “donut-shaped” ring of LFA-ICAM. Compared to the TCR, the attached LFA concentration increases monotonically in time (Fig 3c) and around *t* ≈ 60 *min* saturates the nearly flat membrane. While a similar time evolution is also observed in experiments [3], the choice of scaling makes a direct comparison challenging. This is because one also sees the appearance of a stable circular region of TCR-pMHC surrounded by a donut shaped ring of LFA-ICAM.

**Fig 4:**
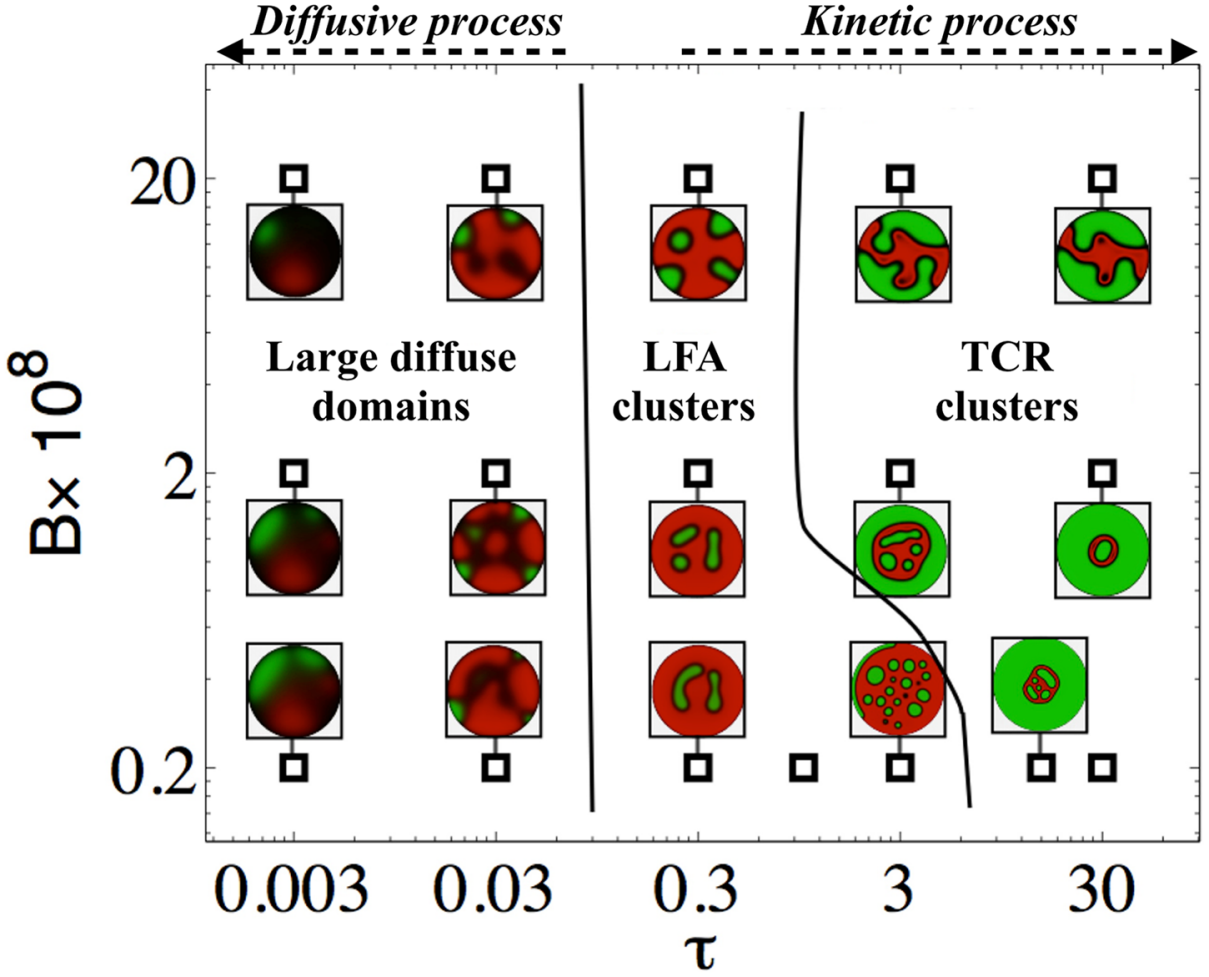
Phase space that characterizes the different regimes of membrane protein patterns as a function of $B=\frac{Eb^{3}}{12\left(1-\nu^{2}\right)\kappa C_{0}L^{4}}$ and $\tau =\frac{\tau_{\mu }}{\tau_{k}}=\frac{\mu }{\tau_{k}C_{0}\kappa l_{2}}$ (see Table 2). The simulations are based on Eqs 1–4 and the patterning is measured at *t* = 40 *min* where a synaptic pattern is typically formed in experiments [2, 3, 20, 21], i.e. in dimensionless units (*L*/*l*_2_)^2^ *t* = 16. Two different protein patterns are identified; large diffuse patches and dispersed kinetic clusters, which are categorized into three regimes. In the diffusional dominated limit (*τ* < 0.3) large diffusive patches are predicted that translocate on the membrane. A transition to a dispersed protein pattern is observed for *τ* > 0.3. In the intermediate regime (0.3 ≤ *τ* ≤ 3), long-lived LFA clusters form on the membrane. When the protein dynamics is an active process (*τ* > 3) micro-scale TCR clusters nucleate and coalesce as they are transported radially forming a central dense pattern. In the kinetic regime we see that the cluster size varies as a function of *B*, similar to our scaling prediction $\approx B^{\frac{1}{4}}$. At equilibrium, all simulations predict a flat membrane with a single protein phase for the case where fluid flux at the edge of the IS is free and the membrane height and number of proteins per membrane area is fixed.

Moving beyond direct comparison with experiments, we turn to a qualitative phase-space of protein patterning characterized by *τ*, *B*, *Pe*, *M*, initial conditions and boundary conditions. Our simulations show that the pattern dynamics are insensitive to variations in *Pe*, *M* and the initial conditions (SI), leaving the scaled *membrane stiffness (*B*)* and the *ratio of time scales (*τ*)* as the main players responsible for variations in the patterns. In Fig 4, we show this in terms of a phase diagram of pattern possibilities illustrated by snapshots of the protein distributions at *t* = 40 *min*, a stage corresponding to a mature IS [2, 3, 10, 20].

Two distinct protein patterns may be identified corresponding to either large diffuse domains or a dispersed micro cluster phase. We can further categorize the latter into two distinct regimes. For *τ* < 0.1 the membrane proteins fail to form an IS and their dynamics are primarily dominated by diffusive fluxes and the results are insensitive to *B*. For *τ* > 0.3 islands of non-overlapping micro-scale protein clusters form different shapes on the membrane. For 0.3 ≤ *τ* ≤ 3 long-lived LFA clusters form at the center and at the edge of the membrane. In this regime, kinetic processes and diffusive fluxes make comparable contributions. By further decreasing the kinetic rate (*τ* > 3) the protein dynamics become hydrodynamically limited with a sharper protein interface. In this regime, a large central domain of TCR with a few internalized LFA micro-clusters form on the membrane, which is surrounded by LFA. We emphasize that at very long times the equilibrium state corresponds to a nearly flat membrane adhesively bound by either TCR or LFA to the bilayer. However, a change in boundary condition that replaces the constant pressure along the edge with a vanishing fluid flux, i.e. ∇*p* ⋅ **n** = 0 where **n** is normal vector at the boundary, leads to an arrested inhomogeneous protein pattern (see S4 Fig and S2 Movie).

Our calculations of the protein patterns show that the formation of IS-like domains only occurs in the hydrodynamically limited regime for *τ* > 0.3. In this regime, protein clusters nucleate at short-time *t* ≈ 1 *min* forming a patchy pattern, with a characteristic cluster size that scales as $l_{c}\approx \left(\frac{B_{m}}{C_{0}\kappa }\right)$ (Eq 5). These micro scale protein clusters move centripetally by the self-generated fluid flow since membrane deformation by protein binding generates flow, which assists sorting and formation of protein domains. Cluster translocation leads to the formation of large protein domains at long times *t* ≈ 30 *min* with the characteristic “donut-shaped” LFA domain that surrounds a central domain dens in TCR (see Fig 4), similar in structure to what is often referred to as a peripheral-SMAC and a central-SMAC in experiments [3, 11, 14, 20, 43].

## Discussion

To get at an accurate description of the spatiotemporal dynamics of protein patterning in the IS we have formulated and solved a minimal mathematical model that account for membrane mechanics, protein binding kinetics and hydrodynamics, while setting the stage for the quantification of passive and active mechanisms in the IS. Our scaling laws for the size of protein clusters, as well as short and long time protein patterning dynamics are corroborated by simulations without ad-hoc physical assumptions. In particular we show that slow dynamics of fluid drainage in the synaptic cleft can account for the time scales of protein patterning. Direct comparison of our computations with experiments [3] suggests that at early times passive dynamics suffices to describe the formation and organization of trans-membrane receptors, and suggests a natural time scale for when active processes come into play. Our passive model of the immune-cell synaptic cleft is a simplification, where we have neglected the mechanisms by which receptor binding generates signaling that triggers internal activity e.g. actomyosin contractility, endo-/exo-cytosis, release of TCR through microvesicles, local recruitment of integrins etc. Since all these effects can influence the patterning dynamics, to challenge our passive physicochemical theory and to help identify the key biophysical process underlying the formation of the IS, we now turn to some experimentally testable predictions.

First, a characteristic spatial scale for membrane deformation is predicted by $l_{c}={\left(\frac{B_{m}}{C_{0}\kappa }\right)}^{\frac{1}{4}}$, where *B*_*m*_ is bending stiffness, *C*_0_ protein number density and *κ* protein stiffness. Since *l*_*c*_ is fairly parameter insensitive, modifying cell membrane rigidity (e.g. using wheat germ agglutinin (WGA) [44]), the protein number density (corralling [46]) or protein stiffness (linker length [45]) would only produce moderate changes in cluster size.

Second, two time scales control the dynamics. At short time protein clusters nucleate $\tau_{c}\approx {\left(\frac{l_{c}}{L}\right)}^{2}\frac{\mu }{C_{0}\kappa }={\left(\frac{l_{c}}{l_{2}}\right)}^{2}\tau_{\mu }$ and at long time and length scales large protein domains form $\tau_{L}\approx {\left(\frac{L}{l_{2}}\right)}^{2}\tau_{\mu }={\left(\frac{L}{l_{2}}\right)}^{2}\frac{\mu }{C_{0}\kappa l_{2}}$. In contrast to the prediction for *l*_*c*_, both *τ*_*c*_ and *τ*_*L*_ are sensitive to changes in protein number density (*C*_0_), protein (*κ*) and membrane stiffness (*B*_*m*_), which can be experimentally changed by corralling, linker-length and WGA and will change these three parameters. Thus, our theory predicts that the time scales for the IS can be changed without much variation in the spatial features.

Third, our numerical simulations predict the formation of IS-like protein domains for *τ*_*μ*_ < *τ*_*k*_, identifying protein kinetics as a critical component in IS formation. This can be tested by changing the adhesion molecules to vary the kinetic time *τ*_*k*_, while *τ*_*μ*_ can be modified by changing the protein number density (corralling) or protein stiffness (linker length).

Fourth, the effective boundary condition at the periphery of the synaptic cleft is found to be a key component in the longevity of the pattern. Simulations allowing fluid flux through the edge of the IS show that the SMACs become unstable at long times. The formation of a tyrosine phosphatase network at the synapse periphery generates additional resistance to fluid drainage and may limit the rate of mass flux. Thus, the proteins at the boundary of the IS may regulate its stability and the disruption of this protein network should affect its longevity.

Fifth, the fluid motion in the membrane gap has hitherto not been quantified. Such experiments may be feasible with quantum dot tracking techniques [47] and may shed new light on the fluid pathway during the patterning. Fluid can either become trapped in thesynaptic cleft, internalized by the cell or escape along its edge.

Sixth, we predict nucleation, translation and sorting of protein clusters in the absence of active processes. Recent observations by [15] of non-immune cells show protein patterning and makes an experimental platform ideal to challenge our spatiotemporal predictions.

Our mathematical model is a minimal and general theoretical skeleton for a description of cell-to-cell interaction, and may be useful more broadly to understand aspects of cell adhesion, communication and motility.

## Supporting Information

S1 Text (PDF)

S1 FigTime history of the attached proteins and the membrane topography as a function of *τ* = [0.03, 0.3, 3.0, 30].
$\tau =\frac{\tau_{\mu }}{\tau_{k}}=\frac{\tau_{\mu }}{\tau_{k}}=\frac{\mu }{\tau_{k}C_{0}\kappa l_{2}}$ is the ratio of the local viscous time (*τ*_*μ*_) and the kinetic time (*τ*_*k*_). *B* = 2 × 10^−8^; the other non-dimensional numbers are given in Table 1. The simulations are based on Eqs 1–4. The color-scale for the density of bonded LFA (green) and TCR (red) proteins is shown in the upper left corner and the scale bar for the membrane height (black-white) is shown in the upper right corner. For *τ* ≪ 1 the dynamics are hydrodynamically limited and no protein clusters are predicted. In contrast, for *τ* > 0.3 clusters of TCR and LFA nucleate at short-time and translocate centripetally at long times forming large protein domains.(TIF)

S2 FigInfluence of protein diffusion, sliding and advection and the initial condition on the predicted numerical results at time *t* = 23 *min* i.e. in dimensionless units ${\left(\frac{l_{2}}{L}\right)}^{2}t^{*}=7.0$.(TIF)

S3 FigTime history of the attached proteins and the membrane topography as a function of off-rate *σ*_*off*_ = ∞ (row 2) and membrane tension Γ = [10^3^–10^5^] (row 3–5) for *B* = 2 × 10^−7^, *τ* = 3.0, *Pe* = 5 × 10^4^ and *M* = 2.0.
$\Gamma =\frac{\gamma }{l_{2}^{2}\kappa C_{0}}$ is the ratio of pressure from the membrane tension and the protein spring pressure. The color-scale for the density of bonded LFA (green) and TCR (red) proteins is shown in the upper left corner and the scale bar for the membrane height (black-white) is shown in the upper right corner. These snapshots in time correspond to dimensionless times ${\left(\frac{l_{2}}{L}\right)}^{2}\times t^{*}=[14,28,71,142]$.(TIF)

S4 FigInfluence of boundary conditions on the IS dynamics (*τ* = 3.0, *B* = 2 × 10^−8^) at times (a) *t* = 10 *min* (${\left(\frac{l_{2}}{L}\right)}^{2}t^{*}=2.8$), (b) *t* = 23 *min* (${\left(\frac{l_{2}}{L}\right)}^{2}t^{*}=7.0$), (c) *t* = 47 *min* (${\left(\frac{l_{2}}{L}\right)}^{2}t^{*}=17$)and (d) *t* = 230 *min* (${\left(\frac{l_{2}}{L}\right)}^{2}t^{*}=69$).At the edge the membrane moves freely so that the torques and forces vanish along the boundary, with no fluid flow and a no-flux boundary condition for the TCR-pMHC and LFA-ICAM proteins. In contrast with the case of the pinned membrane (S1 Fig), which allow in- and out-fluid flow, here the protein pattern is arrested at long times.(TIF)

S1 Movie The dynamics of protein patterning for the case when the fluid flux at the boundary is free to vary, but the pressure is fixed. This causes the pattern to eventually decay.
(MOV)

S2 Movie The dynamics of protein patterning for the case when the fluid flux at the boundary vanishes. This causes the pattern to eventually get arrested.
(MOV)
