# Supplementary material for: Elastohydrodynamics and Kinetics of Protein Patterning in the Immunological Synapse
Source: PLoS Comput Biol. 2015 Dec 23;11(12):e1004481. doi: 10.1371/journal.pcbi.1004481 (PMC4689476; doi:10.1371/journal.pcbi.1004481)
Supplement: S1 Text — (PDF) [file pcbi.1004481.s001.pdf]

S1 Text: Elastohydrodynamics and kinetics of protein patterning in the immunological synapse

Andreas Carlson[1] and L. Mahadevan[1,2,*]

1 School of Engineering and Applied Sciences, Kavli Institute for Bionano Science and Technology, and Wyss Institute, Harvard University, Cambridge, USA
2 Departments of Physics, and Organismic and Evolutionary Biology, Harvard University, Cambridge, USA.

*** lm@seas.harvard.edu**

Problem parameters

Table 1 summarizes material properties that are relevant to the IS synapse, as reported in previous work in the literature and are used as inputs to Eq. 1-4.

Boundary conditions

The boundary condition at the edge of the IS critically affects the final protein pattern since it reflects the role of different biophysical processes associated with membrane deformation, fluid flow and the number of proteins per membrane area. Three different types of boundary conditions for the membrane edge can be prescribed, as given below:

$$\textit{Pinned}:\ \nabla^2 h = 0, h = \textit{constant}, C_1 = C_2 = \textit{constant}$$

$$\textit{Clamped}:\ \nabla h \cdot \mathbf{n} = 0, h = \textit{constant}, C_1 = C_2 = \textit{constant}$$

$$\textit{Free}:\ \nabla^2 h = \nabla^3 h \cdot \mathbf{n} = 0, \nabla \mathbf{J_i} \cdot \mathbf{n} = 0,$$

where $\mathbf{n}$ is the boundary normal. In a slight abuse of notation, we denote *Pinned* and *clamped* as corresponding to fixing the density of proteins at a given height at the membrane edge, and further letting the torque vanish or fixing the angle at the edge. For a *shear and moment free* membrane edge, the edge is free and further we assume that there is no protein flux at the boundary.

Any of these three set of boundary conditions for the membrane height can be prescribed with the additional boundary conditions for the fluid motion that read

$$\textit{Free fluid flux}:\ p = 0$$

$$\textit{No fluid flow } (\mathbf{u} = 0):\ \nabla p \cdot \mathbf{n} = 0.$$

In the case where there are few proteins at the membrane edge, the pressure is prescribed (*free fluid flux*) to allow fluid flow into or out of the membrane gap. If the IS is sealed off by a dense protein network, it is hard for the fluid to escape at the edge and *no fluid flux* is a natural prescription.

In the main text, we have used a pinned membrane with a constant pressure at the edge, allowing mass fluid flux through the boundary of the domain. The use of these boundary conditions is based on experimental observations, where only at late times a

protein network surrounds the IS. One implication of using a *pinned* and *free fluid flux* boundary conditions is that the protein pattern does not stabilize, which can however be arrested by prescribing *no fluid flow* at the free boundary. We note that it is possible to extend the mathematical model to account for a free boundary problem for the location of the edge itself, but we avoid this scenario here as it is not relevant to the dynamics of the IS and generates a significant numerical complication, as a dynamic mesh is needed to track the membrane edge.

Computational methodology

The governing equations (Eq. 1-4) were solved with the open-source finite element toolbox femLego [1]. A first order semi-implicit Euler scheme is used for time marching and all variables are discretized in space using piecewise linear functions. The non-linearity together with the sixth order derivatives in Eq. 2 makes it challenging to solve. Therefore, we decompose this into three equations, for the Laplacian of the height ($\nabla^2 h$), the pressure (p) and the height (h). These three equations are coupled with two additional equations for the proteins, and are solved simultaneously using a Newton iteration method [2].

We performed a convergence study of the 1D results in both time and space; different spatial $\Delta x/L = [0.007, 0.0025, 0.001]$ and temporal $\delta t/\tau_\mu = [100, 400, 1000]$ resolution show no noticeable change in the results. The 2D results presented here use a mesh size ($\Delta x/L = 0.006$) and a time step ($(\frac{l_2}{L})^2 \Delta t^* = 0.02$).

Dependence of dynamics on ratio of hydrodynamic to kinetic time scale τ

To investigate the nature of the spatiotemporal evolution of the trans-membrane proteins we vary τ while keeping all other parameters fixed (Fig. S1). In Fig. S1 we see that when $\tau \ll 1$ the patterns are kinetically limited. Large protein patches nucleate on the membrane that slowly drift by diffusion. In contrast, increasing the role of hydrodynamics by the increase in τ leads to a patchy protein pattern of receptor micro-clusters that are separated by a sharp interface. The clusters move centripetally (see Fig. S1), causing the pattern to coarsen as they coalesce, which leads to the formation of large protein domains. At equilibrium the membrane is nearly flat and saturated by a single protein species.

Parameter sensitivity - diffusion, sliding, initial condition, off-rates and membrane tension

For given initial conditions, protein diffusion, sliding and advection can influence the dynamics of patterning. To quantify the influence of these properties on the resulting protein patterns, we separately turn off these effects. In Fig. S2 we show the results when protein sliding is turned off ($M^{-1} = 0$), in Fig. S2b we show the results when protein advection is turned off ($l_c h_i/L^2 = 0$), and in Fig. S2c we show the results when protein diffusion is turned off ($Pe^{-1} = 0$) (Fig. S2d). What is clear from Fig. S2 is that none of these parameters has any significant contributions in the kinetic regime ($\tau = 3.0$), where macroscopic patterns persist.

In order to determine the influence of diffusion (D) on the resulting dynamics, we varied D over two orders of magnitude (Fig. S2e-g). By changing the diffusion coefficient the value of both Pe and M change. Increasing D makes the interface between the two boundaries more diffuse, while decreasing D makes the boundaries sharper. Comparing the results for $D = 5.0 \times 10^{-13} m^2/s$ and $D = 5.0 \times 10^{-14} m^2/s$ we

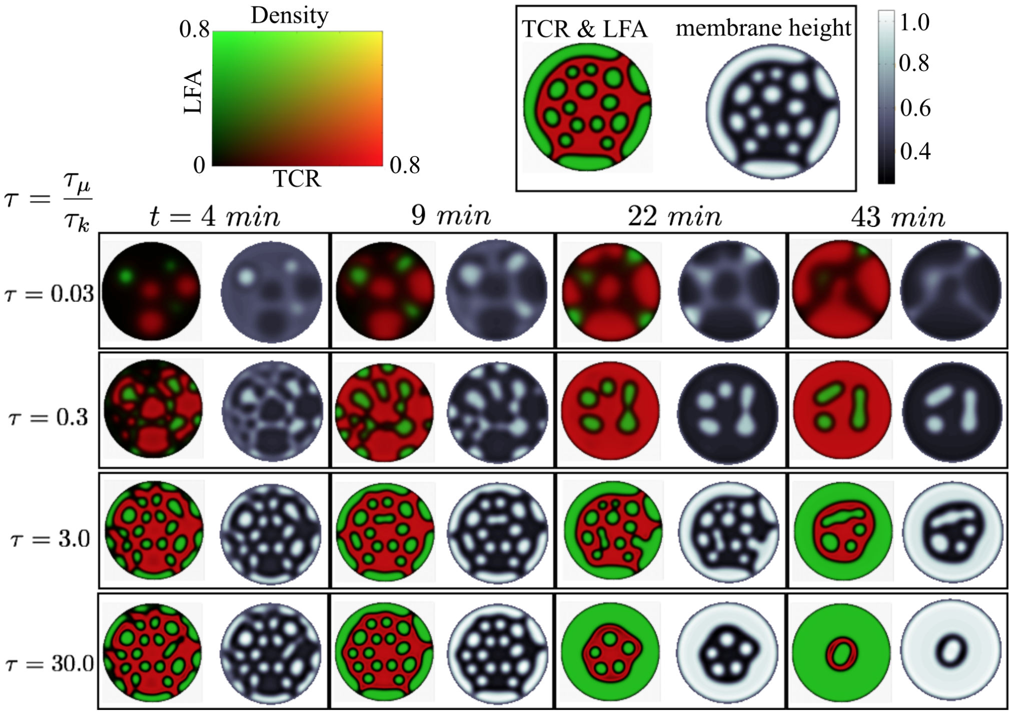

Figure S1. Time history of the attached proteins and the membrane topography as a function of $\tau = [0.03, 0.3, 3.0, 30]$. $\tau = \frac{\tau_\mu}{\tau_k} = \frac{\tau_\mu}{\tau_k} = \frac{\mu}{\tau_k C_0 \kappa l_2}$ is the relative importance between the local viscous time (τ_μ) and the kinetic time rate (τ_k). $B = 2 \times 10^{-8}$ and the other non-dimensional numbers are given in Table 1. The simulations are based on Eq. 1-4. The color-scale for the density of bonded LFA (green) and TCR (red) is shown to the upper left corner and the scale bar for the membrane height (black-white) is shown in the upper right corner. For $\tau \ll 1$ the dynamics are hydrodynamically limited and no protein clusters are predicted. In contrast, for $\tau > 0.3$ clusters of TCR and LFA nucleate at short-time and translocate centripetally at long times forming large protein domains.

notice that besides this quantitative feature, the results are indistinguishable and diffusion does not strongly influence the protein patterns over this range.

To quantify the influence of the initial conditions, we perform simulations with three different initial conditions (Fig. S2h-j). Initially the membrane has six small Gaussian shaped bumps of different widths ($\approx 0.1L$), with an amplitude ($(0.075 - 0.1)l_2$). In the sub-figure to the lower left in Fig. S.2 the bumps on the membrane are inverted compared to the simulation to the lower right. The result presented in the middle sub-figure shows a simulation result with an initial membrane shape with six Gaussian bumps at different positions than shown in the left and right sub-figure. Although the detailed shape of the pattern is slightly influenced by the initial condition, the overall dynamics is robust to these changes.

We have in this work assumed that the kinetic binding and unbinding rates are described by the means passage time over an energy barrier Eq. 4, which leads to an Gaussian distribution for the on/off rates centered around l_i. The off-rates may be a function of the tension in the proteins with a probability of unbinding that increases with the tension up to a given threshold. Eq. 4 generates an effective kinetic rate that takes the form of a double-well, while an off-rate based on the tension in the proteins would remove the two minima and the probability of unbinding approaches a constant

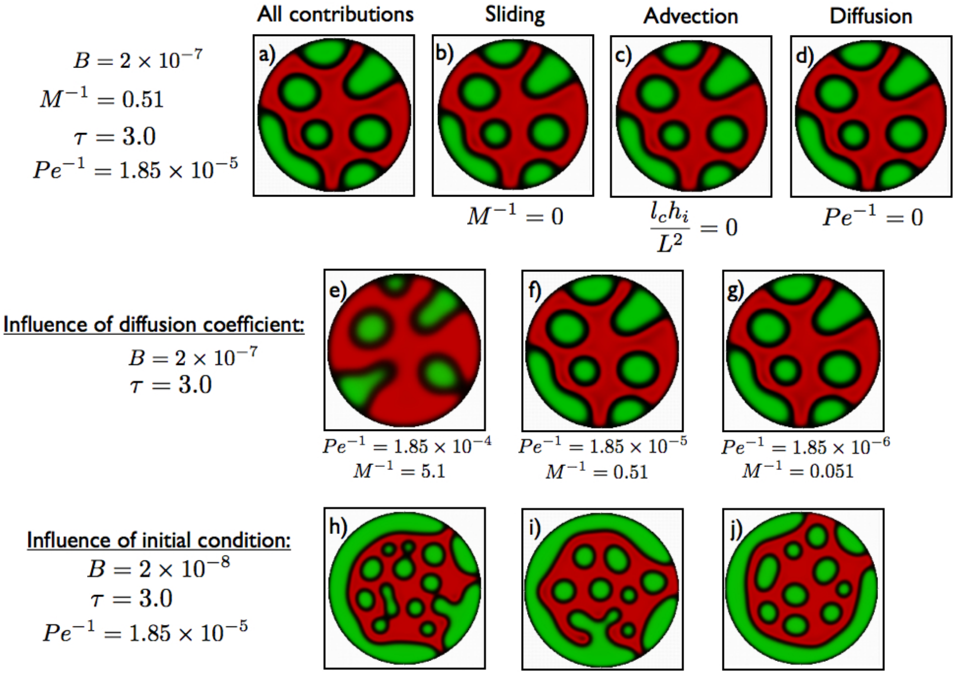

Figure S2. Influence of protein diffusion, sliding and advection and the initial condition on the predicted numerical results at time $t = 23\ min$ i.e. in dimensionless units $(\frac{l_2}{L})^2 t^* = 7.0$.

value as the proteins are further stretched/compressed. The simplest form of a tension based off-rate is to let $\sigma_{off} = \infty$, where the effective rate $(K_i^{on}(l_i) - K_i^{off}(l_i))$ becomes a shift of the gaussian for the on-rate and the probability of unbinding becomes constant for large protein deformation. We have performed additional simulations to verify that our results are not very sensitive to the from of the off-rate, which is demonstrated in the second row in Fig. S.3. Although the detailed shape of the pattern is slightly different, the overall dynamics is robust predicted in the simulation.

Since the membrane has a fluid-like nature, there can also be an influence in the pressure from membrane tension and an additional term $\gamma \nabla^2$ enters into Eq. 1

$$p(x, y, t) = B_m \nabla^4 h - \gamma \nabla^2 h + \kappa C_1 (h - l_1) + \kappa \frac{2 l_1}{l_2} C_2 (h - l_2) \quad \text{(S1)}$$

where γ is the membrane tension $[N/m]$. In the tension dominated limit the length scale for membrane deformation scales as $\left(\frac{\kappa C_0}{\gamma}\right)^{\frac{1}{2}}$. Scaling pressure with the characteristic spring pressure $\kappa l_2 C_0$ yields another dimensionless number $\Gamma = \frac{\gamma}{l_2^2 \kappa C_0}$, which is the ratio between pressure from membrane stretching and the pressure from deforming the protein springs. In Fig. S.3 row 3-5 we demonstrate the influence of membrane tension by varying $\Gamma = [10^3 - 10^5]$ e.g. $\gamma = [4 - 0.04] \times 10^{-3} N/m$ in dimensional units. For $B = 2 \times 10^{-7}$ we note that as $\Gamma < 10^{-5}$ the spatiotemporal dynamics is dominated by membrane bending (Fig. S.3). If the membrane tension is increased, larger protein domains appear $\approx \Gamma^{\frac{1}{2}}$ and if the membrane becomes too stiff the pressure generated by the protein springs is not sufficient to deform the membrane $\Gamma \geq 10^{-2}$.

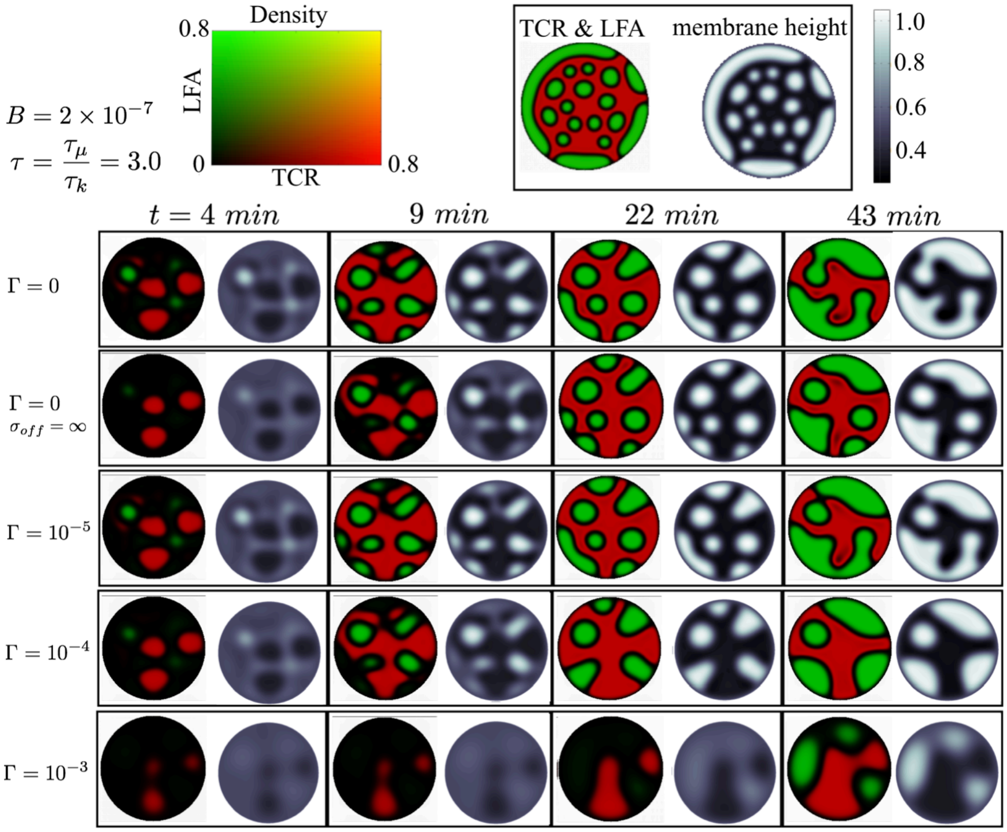

Figure S3. Time history of the attached proteins and the membrane topography as a function of off-rate $\sigma_{off} = \infty$ (row 2) and membrane tension $\Gamma = [10^3 - 10^5]$ (row 3-5) for $B = 2 \times 10^{-7}$, $\tau = 3.0$, $Pe = 5 \times 10^4$ and $M = 2.0$. $\Gamma = \frac{\gamma}{l_2^2 \kappa C_0}$ is the ratio of pressure from the membrane tension and the protein spring pressure. The color-scale for the density of bonded LFA (green) and TCR (red) is shown to the upper left corner and the scale bar for the membrane height (black-white) is shown in the upper right corner. These snapshots in time correspond to the dimensionless times $(\frac{l_2}{L})^2 \times t^* = [14, 28, 71, 142]$.

Table 1. Description of the material parameters that appear in Eq. (1-4).

Description	Notation	Reference
Fluid viscosity	$\mu = 4 \times 10^{-2}\ Pa \cdot s$	
Cell membrane Young's modulus	$E = (0.08 - 80) \times 10^{6} Pa$	
Membrane thickness	$b = 8 \times 10^{9}\ m$	
Poisson ratio	$\nu = 0.5$	[12]
Bending modulus	$B_m = \frac{Eb^3}{12(1-\nu^2)} = 4.5 \times (10^{-21} - 10^{-18})$ J	[7,8]
		[12]
Protein stiffness (Hookean spring)	$\kappa = 1.2 \times 10^{-6} N/m$	[6,7]
		[9]
Equilibrium number density TCR	$C_{1,0} = C_0 = 2 \times 10^{14} m^{-2}$	[3]
Equilibrium number density LFA	$C_{2,0} = 2 \times C_0 = 4 \times 10^{14} m^{-2}$	[3]
Natural TCR-pMHC length	$l_1 = 15\ nm$	[4]
Natural LFA-ICAM length	$l_2 = 45\ nm$	[4]
Membrane protein diffusion coefficient	$D = 5 \times 10^{-13} m/s^2$	[10,11]
Kinetic on-rate	$\tau_k = \tau_1 = \tau_2 = 1.1 \times (10^{-5} - 10^{-1}) s$	
Kinetic off-rate	$\tau^c_{off} = \tau^g_{off} = \tau_k/3$ s	[5]
Cell diameter	$L = 10\ \mu m$	[3]
Hydrodynamic time scale	$\tau_\mu = \frac{\mu}{C_0 \kappa l_2} = 3.7 \times 10^{-3}$ s	
Thermal energy	$k_b T = 4.34 \times 10^{-21} J$	
Distribution width on-rate	$\sigma_{on} = 0.2$	
Distribution width off-rate	$\sigma_{off} = 0.6$	
Pressure scaling	$p_0 = C_0 \kappa l_2 = 10.8\ Pa$	

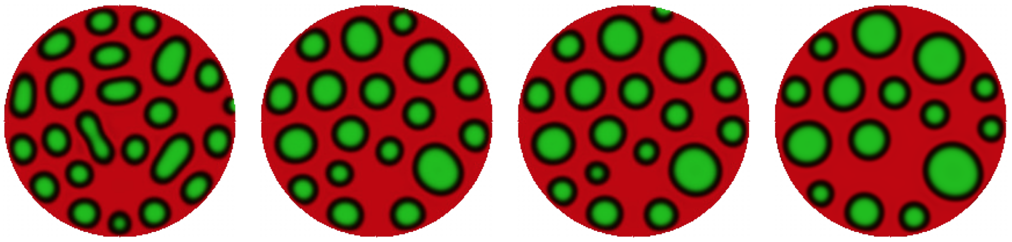

Figure S4. Influence of boundary conditions on the IS dynamics ($\tau = 3.0, B = 2 \times 10^{-8}$) at times (a) $t = 10$ *min* $((\frac{l_2}{L})^2 t^* = 2.8)$, (b) $t = 23$ *min* $((\frac{l_2}{L})^2 t^* = 7.0)$, (c) $t = 47$ *min* $((\frac{l_2}{L})^2 t^* = 17)$and (d) $t = 230$ *min* $((\frac{l_2}{L})^2 t^* = 69)$. At the edge the membrane moves freely using a *shear and moment free* boundary condition, with no fluid flow and a no-flux boundary condition is prescribed for the TCR-pMHC and LFA-ICAM proteins. Contrary to the pinned membrane (Fig. S1), which allow in- and out-fluid flow, the protein pattern is arrested at long times.

Sensitivity to boundary conditions

To illustrate how the boundary condition can affects the simulation results in Fig. S4 we show a sequence of snapshots of a simulation with a membrane that is allowed to move freely at the edge (*shear and moment free*) with *no-flux of proteins* and *no fluid flow*. Comparing these results with the case when proteins are free to diffuse through the boundary, it is clear that the boundary condition affects the protein patterning and serves to arrest the protein pattern at long times.

References

1. Amberg G., R. Tönhardt, and C. Winkler (1999). Finite element simulations using symbolic computing. *Mathematics and Computers in Simulation* **49**, 149–165.

2. Boyanova P. T., M. Do-Quang and M. Neytcheva M. (2012). Efficient preconditioners for large scale binary Cahn-Hilliard models. *Computer Methods in Applied Math* **12**, 1–22.

3. Grakoui A., S. Bromley, C. Sumen, M. Davis, A.S. Shaw, P.M. Allen and M.L. Dustin (1999). The immunological synapse: a molecular machine controlling T cell activation, *Science* **285**, 221.

4. Hartman N. C., J.A. Nye and J.T. Groves. (2009). Cluster size regulates protein sorting in the immunological synapse. *PNAS* **106**, 12729–12734.

5. Figge M. T. and M. Meyer-Hermann (2009). Modeling receptor-ligand binding kinetics in immunological synapse formation. *The European Physical Journal D* **51**, 153–160.

6. Reister E., T. Bihr, U. Seifert and A.S. Smith. (2011). Two intertwined facets of adherent membranes: membrane roughness and correlations between ligand–receptors bonds. *New Journal of Physics* **13**, 02003-1–15.

7. Qi S. Y., J.T. Groves and A.K. Chakraborty. (2001) Synaptic pattern formation during cellular recognition. *PNAS* **98**, 6548–6553.

8. Allard J. F., O. Dushek, D.D. Coombs and P.A. Merwe (2012). Mechanical modulation of receptor-ligand interactions at cell-cell interfaces. *Biophysical Journal* **102**, 1265–1273 .

9. Burroughs N. J. and C. Wülfing. (2002). Differential segregation in a cell-cell contact interface: the dynamics of the immunological synapse. *Biophysical Journal* **83**, 1784–1796.

10. Hsu C.-J., W.T. Hsieh, A. Waldman, F. Clarke, E.S. Huseby, J.K. Burkhardt and T. Baumgart (2012). Ligand mobility modulates immunological synapse formation and T cell activation. *PLoS One* **7**, e32398-1–10.

11. Favier B., N.J. Burroughs, L. Wedderburn and S. Valitutti (2001). TCR dynamics on the surface of living T cells. *International Immunology* **13**, 1525–1532.

12. Simson R., E. Wallraff, J. Faix, J. Niewöhner and G. Gerisch (1998). Membrane bending modulus and adhesion energy of wild-type and mutant cells of Dictyostelium lacking Talin or Cortexillins. *Biophysical Journal* **74**, 514–522.
