# Supplementary figures and images for: Elastohydrodynamics and Kinetics of Protein Patterning in the Immunological Synapse
Source: PLoS Comput Biol. 2015 Dec 23;11(12):e1004481. doi: 10.1371/journal.pcbi.1004481 (PMC4689476; doi:10.1371/journal.pcbi.1004481)

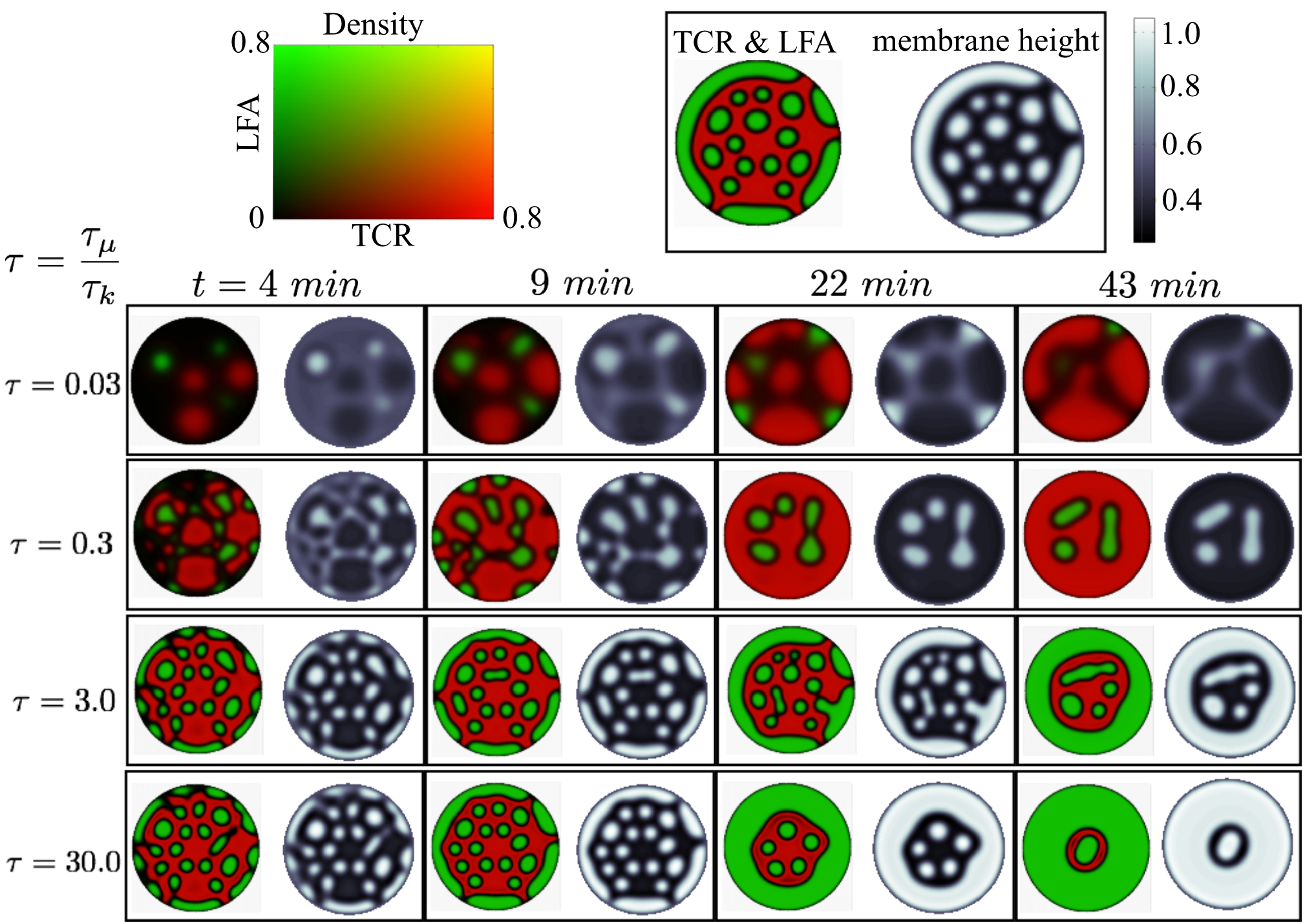

Supplement: S1 Fig — τ=τμτk=τμτk=μτkC0κl2 is the ratio of the local viscous time (τ μ) and the kinetic time (τ k). B = 2 × 10^−8^; the other non-dimensional numbers are given in Table 1. The simulations are based on Eqs 1–4. The color-scale for the density of bonded LFA (green) and TCR (red) proteins is shown in the upper left corner and the scale bar for the membrane height (black-white) is shown in the upper right corner. For τ ≪ 1 the dynamics are hydrodynamically limited and no protein clusters are predicted. In contrast, for τ > 0.3 clusters of TCR and LFA nucleate at short-time and translocate centripetally at long times forming large protein domains. (TIF) [file pcbi.1004481.s002.tif]

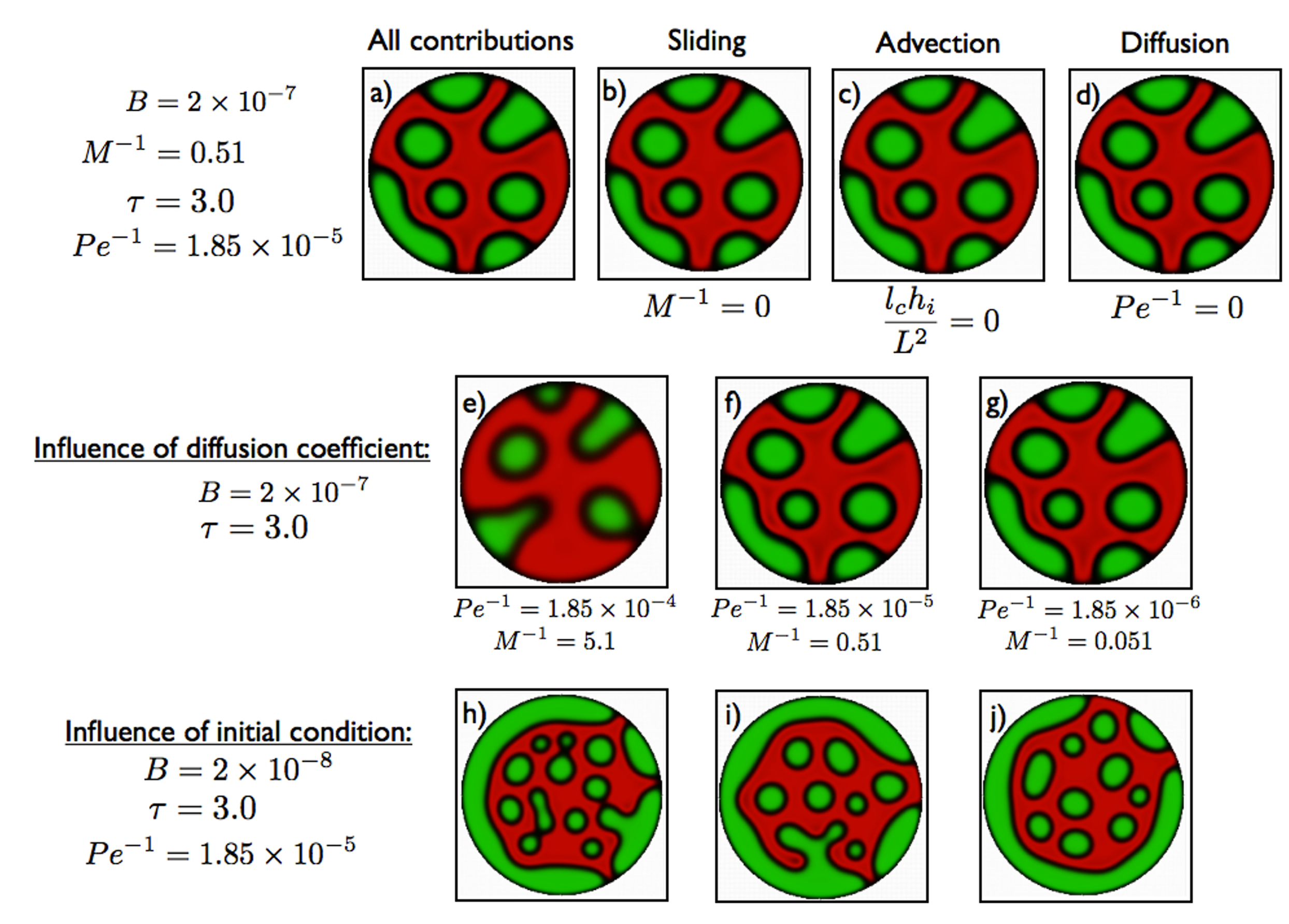

Supplement: S2 Fig — (TIF) [file pcbi.1004481.s003.tif]

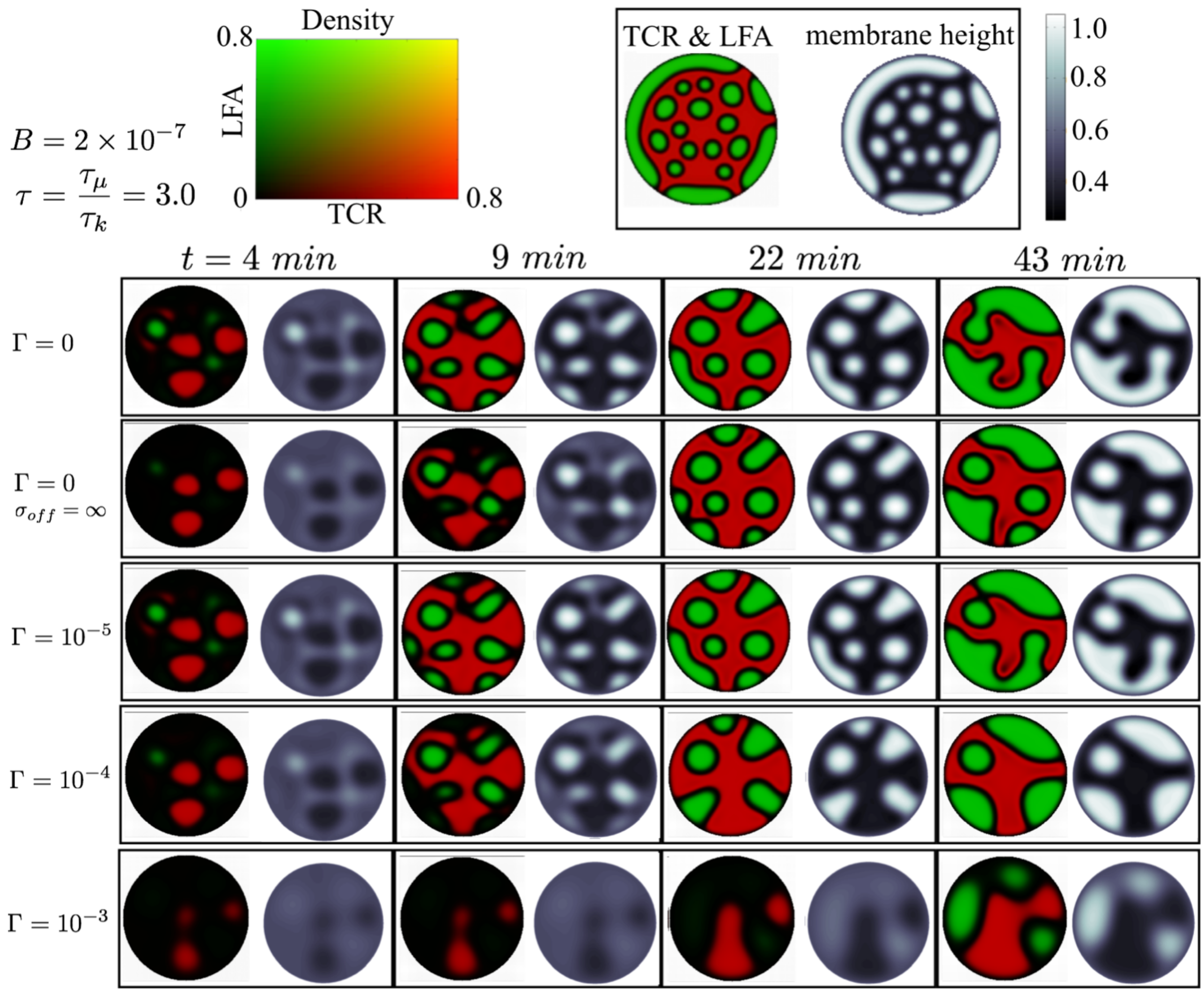

Supplement: S3 Fig — Γ=γl22κC0 is the ratio of pressure from the membrane tension and the protein spring pressure. The color-scale for the density of bonded LFA (green) and TCR (red) proteins is shown in the upper left corner and the scale bar for the membrane height (black-white) is shown in the upper right corner. These snapshots in time correspond to dimensionless times (l2L)2×t*=[14,28,71,142]. (TIF) [file pcbi.1004481.s004.tif]

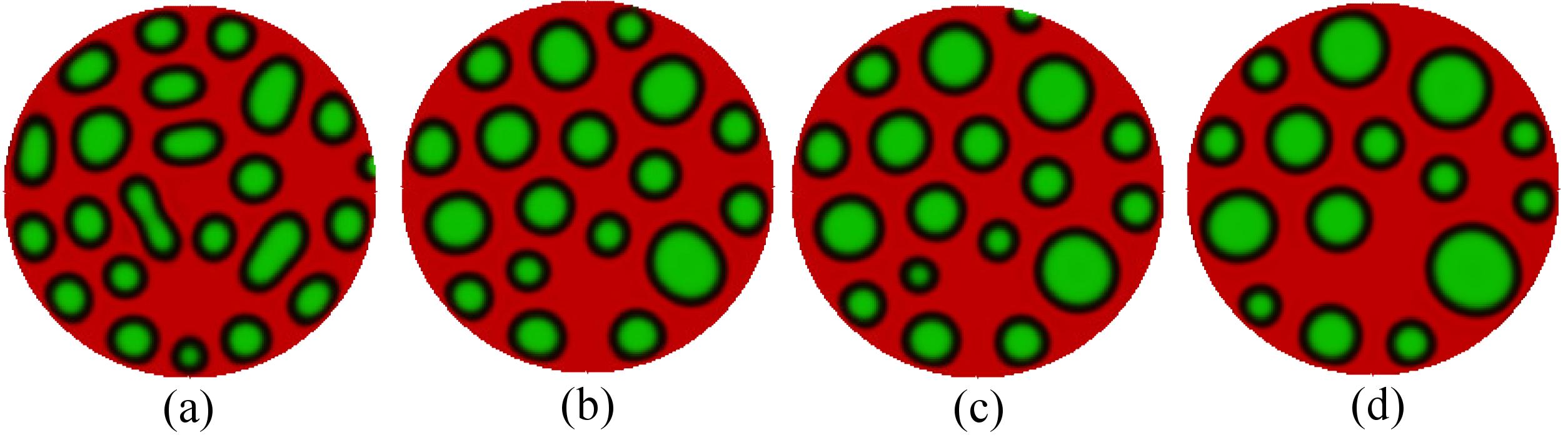

Supplement: S4 Fig — At the edge the membrane moves freely so that the torques and forces vanish along the boundary, with no fluid flow and a no-flux boundary condition for the TCR-pMHC and LFA-ICAM proteins. In contrast with the case of the pinned membrane (S1 Fig), which allow in- and out-fluid flow, here the protein pattern is arrested at long times. (TIF) [file pcbi.1004481.s005.tif]
